# Memory Traces Diminished by Exercise Affect New Learning as Proactive Facilitation

**DOI:** 10.3389/fnins.2020.00189

**Published:** 2020-03-10

**Authors:** Cuicui Li, Rena Li, Chenglin Zhou

**Affiliations:** ^1^Department of Sport Psychology, School of Sport Science, Shanghai University of Sport, Shanghai, China; ^2^Beijing Key Laboratory of Mental Disorders, Beijing Anding Hospital, Capital Medical University, Beijing, China; ^3^Beijing Institute for Brain Disorders, Capital Medical University, Beijing, China

**Keywords:** exercise, forgetting, reversal learning, proactive facilitation, adult-born neurons

## Abstract

Exercise enhances cognitive function through increased neurogenesis but can also cause neurogenesis-induced forgetting. It remains unclear whether the diminished memory traces are completely forgotten. Our goals were to determine whether spatial memory is diminished by exercise, and if so, whether the memory is completely gone or whether only the local details disappear but not the acquired strategy. Two-month-old male C57BL/6J mice were trained on a spatial memory task using the Morris water maze and tested to determine that they had learned the platform location. Another mouse group received no training. Half the mice in each group then exercised on a running wheel, while the other half remained sedentary in home cages. After 4 weeks of this, previously trained mice were tested for their retention of the platform location. All mice were then subjected to the task, but the platform was located in a different position (reversal learning for previously trained mice). We found that exercise significantly facilitated the forgetting of the first platform location (i.e., diminished spatial memory) but also significantly enhanced reversal learning. Compared with mice that received no pre-exercise training, mice that had been previously trained, even those in the exercise group that had decreased recall, showed significantly better performance in the reversal learning test. Activation of new adult-born neurons was also examined. Although newborn neuron activation between groups that had or had not received prior task training was not different, activation was significantly higher in exercise groups than in sedentary groups after the probe test for reversal learning. These results indicated that the experience of pre-exercise training equally facilitated new learning in the sedentary and exercise groups, even though significantly lower memory retention was found in the exercise group, suggesting rule-based learning in mice. Furthermore, newborn neurons equally participated in similar and novel memory acquisition.

## Introduction

Although neurogenesis is one of the primary mechanisms whereby exercise benefits cognitive enhancement, neurogenesis-induced forgetting has also been widely shown (Akers et al., 2014; Gao et al., 2018; Ladron de Guevara-Miranda et al., 2018; Ishikawa et al., 2019). This kind of forgetting was discovered in the phenomenon of “infantile amnesia.” As we all experienced, it is hard for an adult to recall experiences that happened during their infancy, and memories during infancy are more fragile than those during adulthood. Animal studies gave the evidence that infantile rodents had poorer memory retention than adult ones, and this different ability in memory retention was related to the number of newborn neurons in the dentate gyrus (DG) of the hippocampus (Akers et al., 2014; Guskjolen et al., 2016). Hippocampal DG is one of the brain regions maintaining continuous newborn neurons generation after animals matured (Alvarez-Buylla and Lim, 2004). The number of adult-born neurons in DG can be enhanced by the external environment, such as enriched environment and exercise (Nokia et al., 2016; Diederich et al., 2017; Sakalem et al., 2017; Tharmaratnam et al., 2017). Studies showed that exercise could also facilitate the forgetting of previous memory traces through neurogenesis in adult mice, and when exercise-induced neurogenesis is blocked using genetic techniques, exercise-induced forgetting is simultaneously diminished (Akers et al., 2014; Epp et al., 2016). However, numerous studies have shown that exercise could improve memory encoding and consolidation, and this beneficial effect was partially mediated by neurogenesis (Kronenberg et al., 2006; Ahlskog et al., 2011; Alomari et al., 2013; Pontifex et al., 2016; Sakalem et al., 2017). Studies from Epp et al. (2016) partially explained these conflicting results on exercise-induced effects on memories through neurogenesis. They suggested that neurogenesis-induced forgetting would benefit reducing proactive interference (PI) from previous memory traces, therefore further facilitate the following memory encoding with conflict information (Epp et al., 2016). It seems that neurogenesis facilitates the forgetting of remote memories and enhances recent memories. Consistent with this hypothesis, computer modeling studies to simulate the integration of newborn neurons have shown different contributions of neurogenesis to remote and recent memories and have suggested an increase in the forgetting of remote memories with an enhancement for the preservation of recent memories (Weisz and Argibay, 2012).

However, whether these diminished memory traces completely disappear is still unknown. Our knowledge of the external world is always based on the long-term memories we experienced previously. Human studies have shown the benefits of long-term memory for learning new information with similar principle (Gerven et al., 2017; Oberauer et al., 2017; Wang et al., 2017). In addition, rule-based learning (combined cues with an abstract principle) has been reported during associative learning both in human and animal studies (Racht-Delatour and Massioui, 1999; Wills et al., 2011; Maes et al., 2017; Broschard et al., 2019). Compared with remembering only separate cues, rule-based learning is more stable with the passing of time and can adjust to various contexts (Hoffmann et al., 2018). That is, the strategies to solve problems obtained from our experience are more reliable in memory and can facilitate subsequent new learning.

Therefore, in the present study, we hypothesized that the weakened memory traces induced by exercise would still benefit the subsequent new related learning by acting as an abstract strategy. In addition, adult-born neurons were suggested to be involved in both erasing remote and encoding recent memories. It is unclear whether there are any differences between the engagement of adult-born neurons in the acquisition of new memories with or without similar old memory traces. Thus, we further explored the engagement of adult-born neurons in a post-exercise spatial memory task (do or do not receive a pre-exercise spatial memory with similar paradigms). The newborn neurons were labeled with 5-bromo-2′-deoxyuridine (BrdU), and the participation of these neurons in memory acquisition was determined by labeling neurons with immediate early genes (IEGs), which were expressed after stimulation and have been widely used to assess the activation of neurons after memory tasks (Snyder et al., 2009; Belnoue et al., 2011).

## Materials and Methods

### Animals and Experimental Design

The C57BL/6J male mice used in this study were obtained from the Shanghai Laboratory Animal Center (Shanghai, China). All animals were housed in standard home cages in a room with constant temperature (22 ± 2°C) and humidity (50–60%) on a 12-h light/dark cycle (8:00-20:00). Food and water were available freely.

**FIGURE 1:**
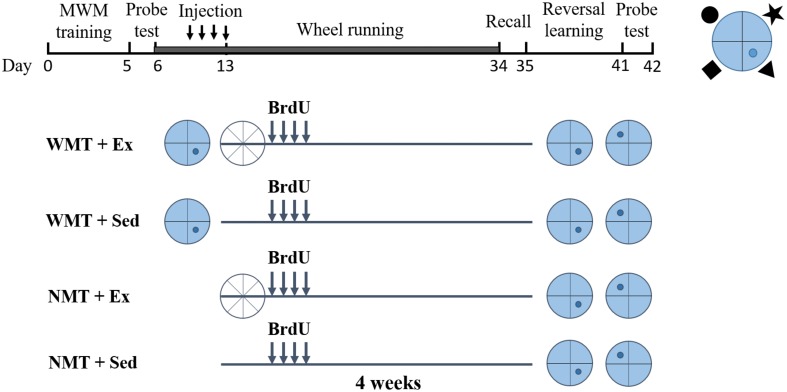
Experimental timeline. Two groups of young adult mice with memory traces (WMT) were trained in the Morris water maze (blue circles with the platform location indicated in darker blue dot), whereas the other two groups with no memory traces (NMT) were not trained but remained in their cages. Half the mice were then subjected to 4 weeks of wheel running exercise (Ex; white circles) and the other half remained sedentary (Sed) in their home cage. To label newborn neurons, 5-bromo-2′-deoxyuridine (BrdU) was injected twice daily into all mice during the last 4 days of the first week of exercise. Following 4 weeks of exercise, a probe test was conducted in the Morris water maze to test recall, followed by a reversal learning task in the maze and a final probe test to examine new memory. A scheme of the position of the platform with respect to the cues used in Morris water maze was shown in the top right corner (the black shapes were the cues).

Mice were used in this study once they were 2 months old (young adults). All mice were handled 5 min each day for 5 days before experiments. To explore whether forgotten memory traces can affect new learning, animals were divided into four groups: (1) with memory traces (WMT) + exercise (Ex), (2) with no memory traces (NMT) + Ex, (3) WMT + sedentary (Sed), and (4) NMT + Sed. During pretesting, animals in the WMT groups were trained in a spatial reference memory task, the MWM, and acquired the spatial memory. Mice in the NMT groups stayed in their home cages, receiving no spatial memory training. Exercise (with sedentary controls) was conducted after the spatial reference memory task and lasted for 4 weeks. Mice in exercise groups were given a running wheel (ENV-044, Med-associates Inc) for voluntary running exercise. The amount of running revolutions were recorded continuously in a subset of cages. Each mouse ran an average of 6.89 km (±0.56 km) per day during the exercise period. After the exercise intervention, the memory retention of the mice in the WMT groups was evaluated on a probe test in the MWM task without the previously hidden platform in the pool. For the same operations among groups during post-exercise training, mice in the NMT groups were also placed in the maze and allowed to swim freely for 60 s. The next day, all mice were subjected to reversal training (Epp et al., 2016; Figure 1).

To label immature newborn cells, BrdU (100 mg/kg; Sigma) was injected intraperitoneally into mice in all groups twice daily for the last 4 days of the first week of exercise (Figure 1).

### Morris Water Maze Task

#### Equipment

The water maze was a circular pool with a diameter of 120 cm. A platform was hidden in one quadrant of the pool 1 cm below the surface of the water, which was made opaque by the addition of milk. The pool was surrounded by a curtain having different shapes located on it in the east, south, west, and north areas. Mice could use these shapes to determine where they were positioned in the maze and thus the location of the hidden platform once they found it. Animal behaviors were tracked and analyzed using ANY-maze software^1^.

#### Pre-exercise Testing

The spatial reference memory task included 5 days of spatial navigation training and 1 day of probe testing. During spatial navigation training, mice learned to ascertain the position of the platform using the visual cues on the curtain during four trials conducted each day. Each trial was started by placing the mouse in one of the quadrants (in semi-random order) facing the wall of the pool. Four start locations were different during each day’s training. And the start quadrant for each day was varied. During testing, the mouse was allowed to swim in the pool until it found the platform or for 60 s. When mice found the platform, they were allowed to stay on the platform for 15 s. If a mouse did not find the hidden platform after 60 s, it was guided to the platform and allowed to remain there for 15 s. Mice were given a probe test 24 h after the last day of training. During the probe test, the previously hidden platform was removed, and mice were allowed to swim for 60 s. The escape latency (the time it takes to find the platform), path length (the distance traveled to find the platform), and swimming speed were calculated during learning. The percent quadrant time (the percentage of time mice spent in a certain quadrant) in the target quadrant (TQ) and in the opposite quadrant (OQ), the average percent quadrant time spent in the two adjacent quadrants (AQs), and the quadrant crossings (the number of crossings in the aforementioned quadrants) during the probe test were collected. In addition, the latency to find the platform and the number of entries in the platform location were also examined.

#### Post-exercise Testing

A probe test was conducted on the first day after 4 weeks of wheel-running exercise. This probe test was used to examine the ability of the mice to recall where the platform had been previously located in the pool. During this probe test, the latency to find the platform and the number of entries in the platform location, the percent quadrant time in the TQ and the OQ, the average percent quadrant time in the two AQs, and the quadrant crossings were examined. The next day, a reversal learning task was initiated. This task also consisted of 5 days of spatial navigation training and a probe test. The only difference between this training/testing and the pre-exercise training/testing was that the position of the hidden platform was moved to a different quadrant. For the 5-day navigation training during reversal learning, the escape latency, path length and swimming speed were collected. Similar to the measures described above, during the probe test of reversal learning, the platform latency and entries, the percent quadrant time, and the quadrant crossings were examined. For the analysis of the effect of memory traces on reversal learning, the original platform (platform during pre-exercise training) crossings and percent quadrant time in the TQ, AQs, and OQ were calculated for the WMT groups during reversal learning. The search strategies among the groups were investigated by examining the percent quadrant time in the corridor connecting the start position with the platform (before the first crossing of the platform location) during the reversal probe test.

### Immunofluorescence

Mice were anesthetized 1.5 h after the end of behavior testing because IEGs are expressed 1-2 h after stimulation. The mice were perfused with 0.9% saline. One hemisphere of each mouse was fixed in 4% paraformaldehyde for 24 h and dehydrated for 30 h in a 30% sucrose solution for cytoprotection. The fixed tissue was then embedded in OCT. The hippocampus was coronally sectioned using a cryostat microtome, with a section thickness of 40 μm.

Hippocampal tissue sections in one hemisphere were taken every 1 in a series of 6 sections (for double labeling) or every 1 in a series of 12 sections (for triple labeling) throughout the entire DG. Free-floating sections were placed in an antigen retrieval solution and heated in a water bath at 80°C for 30 min. The tissue was then placed in 1N HCl and incubated at 37°C for 30 min before being blocked in 3% normal goat serum (containing 0.5% Triton X-100) at room temperature for 1 h. Primary rat monoclonal BrdU antibody (1:300, Abcam, Cat# ab6326, RRID: AB_305426) and mouse monoclonal NeuN antibody (1:500, Millipore, Cat# MAB377, RRID: AB_2298772) with either rabbit anti-c-Fos antibody (1:200, Cell Signaling Technology, Cat# 2250, RRID: AB_2247211) or rabbit anti-EGR1 antibody (1:300, Cell Signaling Technology, Ca.# 4153, RRID: AB_2097038) were incubated with the tissue overnight at 4°C. The next day, the secondary antibodies goat anti-mouse Alexa Fluor 488 (1:500, Invitrogen), goat anti-mouse Alexa Fluor 594 (1:500, Invitrogen), or goat anti-mouse Alexa Fluor 647 (1:500, Invitrogen) were incubated as appropriate with the tissue for 1 h at room temperature to enable visualization of the primary antibodies.

The quantification of double/triple-labeled cells was performed using a confocal microscope (Zeiss, Jena, Germany) equipped with a 63 × oil-immersion objective. All potential labeled cells through the SGZ-GCL from multiple sections were scanned with a 1 μm interval. To estimate the total number of adult-born neurons for DG (bilaterally), the number of BrdU/NeuN immunoreactive cells was tallied and multiplied by 12 (Tronel et al., 2015). The proportion of the activated adult-born neurons was estimated by triple labeled by BrdU/NeuN/IEGs, and 100-200 adult-born cells were analyzed per mouse.

### Statistical Analysis

All data are graphed as means ± standard error of the mean (SEM). Behavioral data, such as escape latency, path length, and swimming speed, during pre-exercise and reversal learning were analyzed using two-way or three-way repeated-measures analysis of variance (ANOVA). Behavioral parameters during the probe test, such as percent quadrant time and quadrant crossings, were analyzed using two-way ANOVA. The immunofluorescence data were analyzed using two-way ANOVA. The criterion for statistical significance was α = 0.05. All analyses were carried out using SPSS, version 17.0 (StataCorp).

## Results

### Exercise Reduces Memory Retention

**FIGURE 2:**
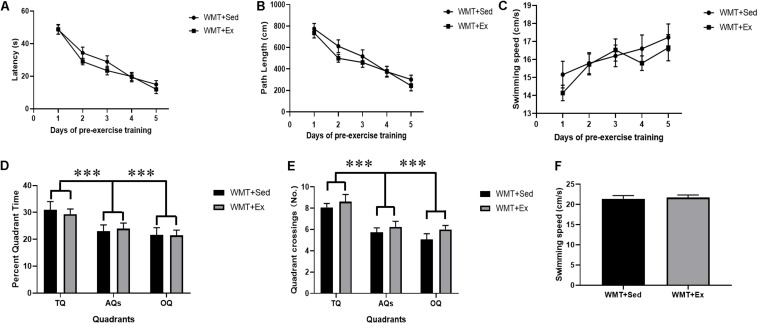
Mice successfully acquired the spatial memory during pre-exercise learning. **(A,B)** Two-way repeated-measures ANOVA shows no difference between groups of mice that were sedentary with memory traces (WMT + Sed) and those with memory traces plus exercise (WMT + Ex) in their performance during pre-exercise training. **(C)** Comparison of swimming speed between the WMT + Sed and WMT + Ex groups during pre-exercise training. There is no difference in the swimming speed between groups. **(D,E)** Two-way ANOVA shows no difference between groups of mice in their performance during pre-exercise probe test, and mice shows significant larger time and more entries in the target quadrant (TQ) than that in the adjacent quadrants (AQs) and opposite quadrant (OQ). **(F)** Comparison of swimming speed between the WMT + Sed and WMT + Ex groups during probe test. There is no difference in the swimming speed between groups. Data are graphed as means ± SEM (*n* = 13) and analysis used two-way repeated ANOVA **(A–C)** and two-way ANOVA **(D,E)**. ^∗∗∗^*p* < 0.001.

Two-way repeated-measures ANOVA was used to analyze latency and path length during pre-exercise training. All mice in the WMT + Sed and WMT + Ex groups exhibited a similar ability to locate the platform during 5 days of learning (for latency: day main effect, *F*_(__4_,_96__)_ = 48.387, *p* < 0.001; group main effect, *F*_(__1_,_24__)_ = 1.73, *p* > 0.05; day × group interaction: *F*_(__4_,_96__)_ = 0.557, *p* > 0.05; for path length: day main effect: *F*_(__4_,_96__)_ = 26.316, *p* < 0.001; group main effect: *F*_(__1_,_24__)_ = 4.061, *p* > 0.05; day × group interaction: *F*_(__4_,_96__)_ = 0.344, *p* > 0.05) (Figures 2A,B). Swimming speeds between groups were examined using two-way repeated measures and the results showed increased swimming speeds for all mice during 5 days of water maze training, and no difference was found between groups (day main effect: *F*_(__4_,_96__)_ = 5.645, *p* < 0.001; group main effect: *F*_(__1_,_24__)_ = 0.486, *p* > 0.05; day × group interaction: *F*_(__4_,_96__)_ = 0.584, *p* > 0.05) (Figure 2C). During probe test, the percentage of time mice explored in different quadrants (TQ, AQs, and OQ) and quadrant crossings between groups were analyzed using two-way ANOVA. Mice in both groups showed significantly higher percentage time in the TQ compared with the other quadrants (quadrant main effect: *F*_(__2_,_98__)_ = 7.817, *p* = 0.001; group main effect: *F*_(__1_,_98__)_ = 0.031, *p* > 0.05; quadrant × group interaction: *F*_(__2_,_98__)_ = 0.182, *p* > 0.05) (Figure 2D). Similarly, mice in both groups performed a larger number of crossings in the TQ compared with the other quadrants (quadrant main effect: *F*_(__2_,_98__)_ = 19.455, *p* < 0.001; group main effect: *F*_(__1_,_98__)_ = 3.077, *p* > 0.05; quadrant × group interaction: *F*_(__2_,_98__)_ = 0.127, *p* > 0.05) (Figure 2E). The swimming speeds during probe test were also examined using Student’s *t* test and no difference was found between the WMT + Sed and WMT + Ex groups (*t*_24_ = 0.366, *p* > 0.05) (Figure 2F).

**FIGURE 3:**
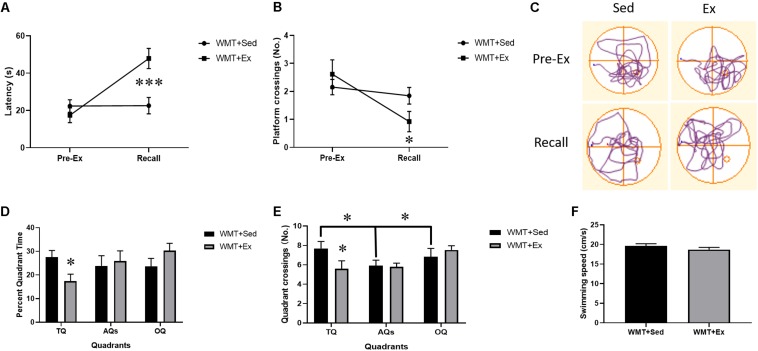
Exercise facilitates the diminishment of acquired memory traces. **(A,B)** Comparison of memory retention (pre-exercise probe vs. recall) between the WMT + Sed and WMT + Ex groups after 4 weeks of housing in standard cages (Sed) or in cages equipped with a running wheel (Ex). There is a significant increase in the latency to find the platform location and decrease in the number of entries in the platform when compared with that during the pre-exercise probe test in the WMT + Ex group, whereas no significant change is detected for the WMT + Sed group. **(C)** Trajectories of mice exploring the maze during the pre-exercise probe and recall tests. **(D)** The percentage of time mice spend in different quadrants during the probe test of recall. Exercise significantly reduces mice exploration in the target quadrant (TQ) when comparing with sedentary group. No difference is found in the exploration in different quadrants (target: TQ, adjacent: AQs and opposite quadrants: OQ) for both groups. **(E)** The number of quadrant crossings during recall probe test. Mice in the WMT + Ex group show decreased the TQ crossings when comparing with mice in the WMT + Sed group. Mice in the WMT + Sed group exhibit more entries in the TQ than AQs and TQ. **(F)** Comparison of swimming speed between the WMT + Sed and WMT + Ex groups during probe test. There is no difference in the swimming speed between groups. Data are graphed as means ± SEM (*n* = 13) and were analyzed using two-way repeated ANOVA **(A,B)** and two-way ANOVA (D, E). ^∗^*p* < 0.05, ^∗∗∗^*p* < 0.001.

To explore the effects of exercise on memory retention, we compared the latency to find the virtual platform and the number of crossings in the original platform location in the first probe test (platform removed) with that during the recall test (i.e., the probe test conducted after 4 weeks of wheel-running exercise) using a 2 (probe test: Pre-Ex, recall) × 2 (group: WMT + Sed, WMT + Ex) repeated-measures ANOVA. The results showed that 4 weeks’ exercise intervention significantly increased the platform latency (probe test × group interaction: *F*_(__1_,_24__)_ = 13.419, *p* = 0.001; simple effects test: WMT + Ex group, *t*_12_ = -5.127, *p* < 0.001) and decreased the number of crossings in the platform location (probe test × group interaction: *F*_(__1_,_24__)_ = 4.439, *p* < 0.05; simple effects test: WMT + Ex group, *t*_12_ = 2.999, *p* < 0.05) (Figures 3A–C). We also compared the percentage of time mice searched in different quadrats (TQ, AQs, OQ) and the number of crossings in these quadrants between the WMT + Sed and WMT + Ex groups during recall. Two-way ANOVA was used in this analysis. The results showed that mice in the WMT + Ex group decreased the percentage of time spent in the TQ when compared with mice in the WMT + Sed group, while no difference was found among quadrants (quadrant × group interaction: *F*_(__2_,_98__)_ = 3.469, *p* < 0.05; simple effects test: WMT + Sed vs. WMT + Ex in TQ, *t*_24_ = -2.478, *p* < 0.05) (Figure 3D). In addition, mice in the WMT + Ex group exhibited fewer crossings in the TQ compared with mice in the WMT + Sed group. Mice in the WMT + Sed exhibited more entries in the TQ than the other quadrants (quadrant × group interaction: *F*_(__2_,_98__)_ = 5.038, *p* < 0.01; simple effects test: WMT + Sed vs WMT + Ex in TQ, *t*_24_ = −2.694, *p* < 0.05; different quadrants in WMT + Sed group, *F*_(__2_,_51__)_ = 3.598, *p* < 0.05) (Figure 3E).

To exclude the effect of swim speed on the performance in the water maze, we compared the swim speed during the recall test between the WMT groups with or without running exercise. No difference was found between the WMT + Sed and WMT + Ex groups (*t*_24_ = −1.124, *p* > 0.05) (Figure 3F).

### Experience Equally Facilitates Associative Learning Despite Significantly Lower Memory Retention in Exercise Group

**FIGURE 4:**
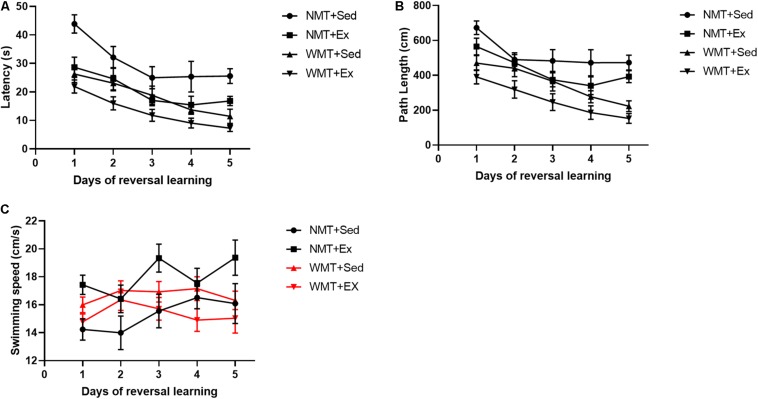
Weakened memory traces induced by exercise still facilitate new learning. **(A,B)** Prior experience in the Morris water maze facilitates later reversal learning in the maze in both the sedentary (Sed) and exercise (Ex) groups during 5 days acquisition phase. And no matter mice in with no memory trace (NMT) or with memory trace (WMT) groups, mice engaged in exercise training performed much better than that in sedentary during new learning. **(C)** Swimming speed during reversal learning. Swimming speed increases during the 5 days of learning in NMT groups. Exercise increases mice swimming speed in the NMT groups. Data analysis used three-way repeated ANOVA.

To investigate whether spatial memory traces decreased by exercise have any effects on new spatial learning, we compared the latencies and path lengths to reach the target platform during the 5 days of post-exercise training among the groups using three-way repeated ANOVA. The results indicated that exercise (Ex) and experience (WMT) both had significant facilitation on new spatial learning with decreased target platform latencies (exercise main effect: *F*_(__1_,_38__)_ = 21.389, *p* < 0.01; experience main effect: *F*_(__1_,_38__)_ = 32.947, *p* < 0.01; exercise × experience interaction: *F*_(__1_,_38__)_ = 1.785, *p* > 0.05) and reduced path lengths (exercise main effect: *F*_(__1_,_38__)_ = 10.036, *p* < 0.01; experience main effect: *F*_(__1_,_38__)_ = 32.246, *p* < 0.001; exercise × experience interaction: *F*_(__1_,_38__)_ = 1.785, *p* > 0.05) during 5 days’ reversal learning (Figures 4A,B). Swimming speeds were also examined during reversal learning using three-way repeated-measures ANOVA. The results showed that the swimming speed in the NMT groups increased during the 5 days of learning, whereas there were no significant changes in the WMT groups (day × experience interaction: *F*_(__4_,_152__)_ = 3.898, *p* < 0.01; simple effects test: in the NMT group, day main effect, *F*_(__4_,_96__)_ = 3.534, *p* < 0.05; in the WMT groups, day main effect, *F*_(__4_,_96__)_ = 1.793, *p* > 0.05). In addition, exercise increased the swimming speed of mice only in the NMT groups (exercise × experience interaction: *F*_(__1_,_38__)_ = 8.916, *p* < 0.01; simple effects test: in the NMT group, exercise main effect, *F*_(__1_,_14__)_ = 6.285, *p* < 0.05; in the WMT groups, exercise main effect, *F*_(__1_,_24__)_ = 2.546, *p* > 0.05) (Figure 4C).

**FIGURE 5:**
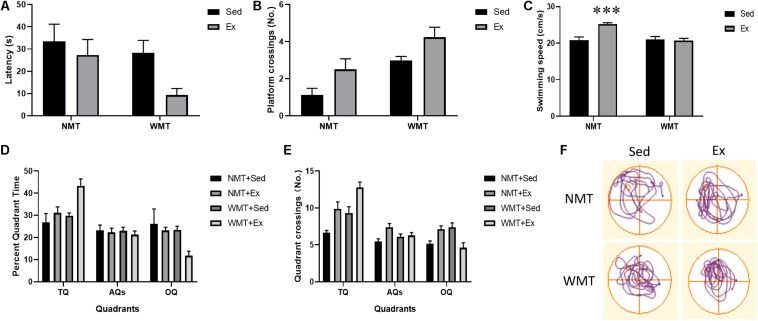
Weakened memory traces induced by exercise facilitate consolidation of new memory. **(A,B)** Experience and exercise both facilitate decreasing target platform latency and enhance the number of crossings in the platform location during the final probe test. **(C)** Swimming speed during the final probe test. Exercise increased mice swimming speed in the NMT groups. **(D,E)** Experience and exercise increase the percent quadrant time and number of crossings in the target quadrant (TQ). Similarly, both experience and exercise decreased the percent quadrant time in the opposite quadrant (OQ). Exercise increases the number of OQ crossings in the NMT groups, whereas it decreases the number of OQ crossings in the WMT groups. Exercise also increases adjacent quadrants (AQs) crossings only in the NMT groups, with no effect on the percent quadrant time in AQs in both the NMT and WMT groups. **(F)** Trajectories of mice exploring the maze during the final probe test. Data are graphed as means ± SEM (*n* = 8–13); Data analysis used two-way ANOVA. ^∗∗∗^*p* < 0.001.

During the reversal probe test, we compared the escape latency and platform entries among the different groups using two-way ANOVA. Exercise and experience both showed facilitation of searching for the platform location with decreased latency (exercise main effect: *F*_(__1_,_38__)_ = 4.554, *p* < 0.05; experience main effect: *F*_(__1_,_38__)_ = 4.583, *p* < 0.05; exercise × experience interaction: *F*_(__1_,_38__)_ = 1.112, *p* > 0.05) and increased number of platform entries (exercise main effect: *F*_(__1_,_38__)_ = 6.463, *p* < 0.05; experience main effect: *F*_(__1_,_38__)_ = 10.112, *p* < 0.01; exercise × experience interaction: *F*_(__1_,_38__)_ = 0, *p* > 0.05) (Figures 5A,B). The swimming speeds among the groups during the probe test were examined using two-way ANOVA. Exercise increased the swimming speed only in the NMT group (exercise × experience interaction: *F*_(__1_,_38__)_ = 9.216, *p* < 0.01; simple effects test: NMT + Sed vs. NMT + Ex, *t*_14_ = 4.734, *p* < 0.001; WMT + Sed vs. WMT + Ex, *t*_24_ = -0.259, *p* > 0.05) (Figure 5C). In addition, we investigated the percentage of time spent in the various quadrants among the groups. The results showed that exercise and experience both increased the time mice spent in the TQ during the 60 s exploration of the probe test (exercise main effect: *F*_(__1_,_38__)_ = 10.239, *p* < 0.01; experience main effect: *F*_(__1_,_38__)_ = 6.817, *p* < 0.05; exercise × experience interaction: *F*_(__1_,_38__)_ = 2.913, *p* > 0.05) and reduced the percent quadrant time in the OQ (exercise main effect: *F*_(__1_,_38__)_ = 5.128, *p* < 0.05; experience main effect: *F*_(__1_,_38__)_ = 5.257, *p* < 0.05; exercise × experience interaction: *F*_(__1_,_38__)_ = 1.765, *p* > 0.05). No difference was found in the time spent in the AQs for mice among the different groups (Figure 5D). The quadrant crossings were also examined, and the results showed that exercise and experience both increased the TQ crossings (exercise main effect: *F*_(__1_,_38__)_ = 14.524, *p* < 0.001; experience main effect: *F*_(__1_,_38__)_ = 10.028, *p* < 0.01; exercise × experience interaction: *F*_(__1_,_38__)_ = 0.014, *p* > 0.05). When analyzing the OQ crossings, we found that exercise increased the OQ crossings in the NMT groups, but decreased the OQ crossings in the WMT groups (exercise main effect: *F*_(__1_,_38__)_ = 0.348, *p* > 0.05; experience main effect: *F*_(__1_,_38__)_ = 0.037, *p* > 0.05; exercise × experience interaction: *F*_(__1_,_38__)_ = 13.385, *p* = 0.001; simple effects test: NMT + Sed vs. NMT + Ex, *t*_24_ = 2.576, *p* < 0.05, WMT + Sed vs. WMT + Ex, *t*_24_ = −3.079, *p* < 0.01). For the AQ crossings, exercise showed an enhanced effect on the crossing numbers in the AQs for both the NMT and WMT groups (exercise main effect: *F*_(__1_,_80__)_ = 5.861, *p* < 0.05; experience main effect: *F*_(__1_,_80__)_ = 0.281, *p* > 0.05; exercise × experience interaction: *F*_(__1_,_80__)_ = 3.935, *p* > 0.05) (Figures 5E,F).

### Exercise Facilitates the Development of a Direct Search Strategy in the Water Maze

**FIGURE 6:**
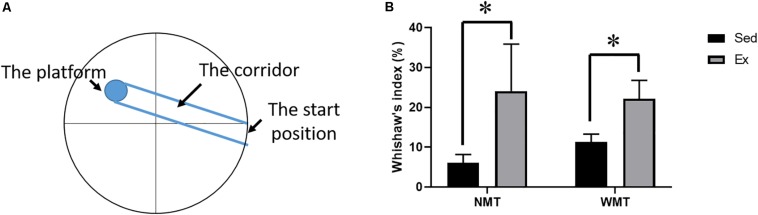
Search strategies in different groups. **(A)** Scheme of maze configuration showing the corridor. **(B)** Different search strategies among groups. Mice in exercise (Ex) groups, including NMT + Ex and WMT + Ex groups, prefer a direct search strategy with more percent quadrant time in the corridor (connecting the start position with the platform) before the first entry to the platform. Data are graphed as means ± SEM (*n* = 8–13); Data analysis used two-way ANOVA. ^∗^*p* < 0.05.

To investigate the search strategies in the different groups during post-exercise learning, we compared the Wishaw’s index (the percent time in the corridor connecting the start position with the platform) using two-way ANOVA. The results showed that mice in both NMT + Ex and WMT + Ex groups preferred the direct search strategy with more percent quadrant time in the corridor [exercise main effect: *F*_(__1_,_38__)_ = 6.551, *p* < 0.05; experience main effect: *F*_(__1_,_38__)_ = 0.09, *p* > 0.05; exercise × experience interaction: *F*_(__1_,_38__)_ = 0.4, *p* > 0.05] (Figures 6A,B).

### Memory Traces Differentially Affect New Spatial Learning in Sedentary and Exercise Groups

**FIGURE 7:**
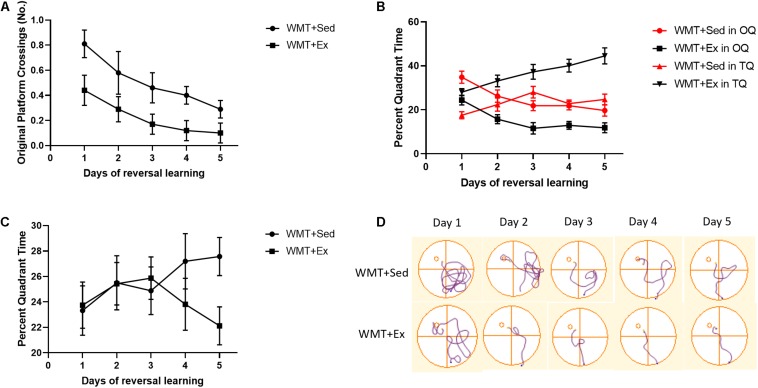
Lower learning efficiency in sedentary mice may be attributable to maintained memory traces. **(A)** Comparison of the number of crossings over the original platform (the platform during pre-exercise training) between groups of mice that were sedentary with memory traces (WMT + Sed) and mice with memory traces that exercised (WMT + Ex). Mice in the WMT + Sed group show significantly higher numbers of crossings over the original platform location during 5 days of reversal learning than mice in the WMT + Ex group. **(B)** Comparison of the percentage of time spent in the new target quadrant (TQ) and opposite quadrant (OQ) between the WMT + Sed and WMT + Ex groups during reversal learning. Both groups increase exploration time in the TQ and decrease exploration time in the OQ. However, the difference in exploration time between the TQ and the OQ in the WMT + Sed group is statistically significant only on the first day of reversal learning. By contrast, mice in the WMT + Ex group spend significantly more time in the TQ and less time in the OQ during the last 4 days of reversal learning, but not on the first day. **(C)** Comparison of the percentage of time spent in the adjacent quadrants (AQs) between the WMT + Sed and WMT + Ex groups during reversal learning. Both groups spent similar time in AQs during 5 days of learning. **(D)** The trajectories of mice exploring the maze during the 5 days of reversal learning. Data are graphed as means ± SEM (*n* = 13) and were analyzed using two-way repeated-measures ANOVAs.

We investigated how former memory traces affect associated new learning. To this end, we first compared the number of crossings over the former platform location (after the platform was moved to a new location) between the WMT + Sed and WMT + Ex groups during 5 days of reversal learning using a two-way repeated-measures ANOVA. The results showed that both groups decreased the number of crossings over the former platform location with reversal learning. However, the number of crossings was significantly lower in the WMT + Ex group than in the WMT + Sed group throughout the learning process (day main effect: *F*_(__4_,_96__)_ = 3.616, *p* < 0.01; group main effect: *F*_(__1_,_24__)_ = 9.548, *p* < 0.01; day × group interaction: *F*_(__4_,_96__)_ = 0.25, *p* > 0.05) (Figure 7A).

We then compared the WMT + Sed and WMT + Ex groups for the percentage of time spent in the opposite quadrant where the platform had been originally located, that is, the OQ, and the new platform location, that is, the new target quadrant (TQ). We found that exercise facilitated acquisition of the new TQ location during the 5 days of reversal training in the WMT + Ex group, with increased time in the TQ (group main effect: *F*_(__1_,_24__)_ = 44.337, *p* < 0.001; day × group interaction: *F*_(__4_,_96__)_ = 1.794, *p* > 0.05, two-way repeated-measures ANOVA) and decreased time in the OQ (group main effect: *F*_(__1_,_24__)_ = 21.304, *p* < 0.001; day × group interaction: *F*_(__4_,_96__)_ = 0.161, *p* > 0.05, two-way repeated-measures ANOVA). However, when we compared the difference in the time spent in the new TQ and OQ between the WMT + Sed and WMT + Ex groups, we discovered that the two groups were using different strategies. During reversal training, the percentage of time spent in the new TQ increased and the percentage of time spent in the OQ decreased for mice in the WMT + Sed group (quadrant × day interaction: *F*_(__4_,_96__)_ = 9.728, *p* < 0.01, two-way repeated-measures ANOVA); but the percentage of time spent in the TQ was significantly different (lower) from that spent in the OQ only on the first day of training (*t*_24_ = 5.452, *p* < 0.01). By contrast, mice in the WMT + Ex group spent the same percentage of time in the two quadrants on the first day of reversal training, but time spent in the TQ increased with training days while time spent in the OQ decreased with training (quadrant main effect: *F*_(__1_,_24__)_ = 105.67, *p* < 0.001; quadrant × day interaction: *F*_(__4_,_96__)_ = 11.244, *p* < 0.001, two-way repeated-measures ANOVA) (Figure 7B).

To confirm that the lower learning efficiency for mice in WMT + Sed groups was only due to the longer exploration in OQ, we also compared the exploration time in AQs between the WMT + Sed and WMT + Ex groups during 5 days of reversal learning. The results showed that WMT + Sed and WMT + Ex groups spent similar time in the AQs during the 5 days of reversal learning (group main effect: *F*_(__1_,_50__)_ = 2.003, *p* > 0.05; day × group interaction: *F*_(__4_,_200__)_ = 1.092, *p* > 0.05, two-way repeated-measures ANOVA) (Figures 7C,D).

### Exercise Enhances Hippocampal Neurogenesis

**FIGURE 8:**
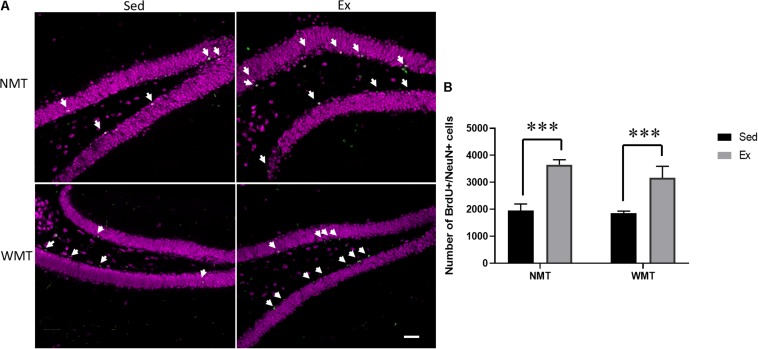
Exercise facilitates adult-born neurons. **(A)** Representative images of adult-born neurons labeled with 5-bromo-2′-deoxyuridine (BrdU; indicated with arrows) and NeuN in the hippocampal dentate gyrus (DG) of mice in the sedentary (Sed) and exercise (Ex) groups. NMT represents no memory traces; WMT, with memory traces; scale bar, 50 μm. **(B)** Four weeks of wheel running enhances neurogenesis in the DG. Data are graphed as means ± SEM (n = 3–4); Data analysis used two-way ANOVA. ^∗∗∗^*p* < 0.001.

The number of adult-born neurons labeled with BrdU/NeuN was compared among the groups using a 2 (experience: NMT, WMT) × 2 (group: Sed, Ex) two-way ANOVA. The results showed that there was a significant main effect of group between the exercise and sedentary groups (*F*_(__1_,_11__)_ = 29.207, *p* < 0.001) (Figure 8).

### Exercise Enhances the Activation of Adult-Born Neurons in New Associative Learning

**FIGURE 9:**
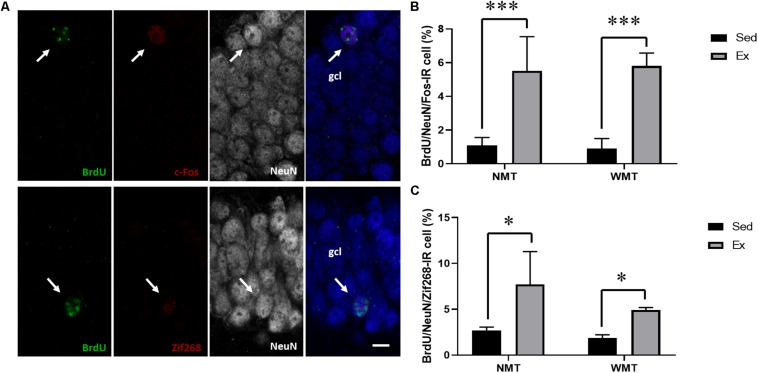
Spatial memory induces activation of IEGs in adult-born neurons. **(A)** Representative images of adult-born neurons activated during the probe test of reversal learning. The fourth column is the merged image. Arrows indicate the respective labeling; scale bar, 10 μm. **(B,C)** Percentage of adult-born neurons expressing c-Fos or Zif268 in the granular cell layer (gcl) of the hippocampal dentate gyrus. Ex indicates exercise; NMT, mice with no memory traces; WMT, mice with memory traces; and Sed, sedentary. Data are graphed as means ± SEM (*n* = 3–4). Data analysis used two-way ANOVA. ^∗^*p* < 0.05, ^∗∗∗^*p* < 0.001.

immediate early genes, such as c-Fos and Zif268 (also known as early growth response 1 or Egr1), are commonly used to investigate the activation of neurons during learning and other stimulation. Here, we explored the participation of the adult-born neurons in new spatial learning under different conditions by analyzing the proportion of newborn neurons labeled with BrdU/NeuN/c-Fos or BrdU/NeuN/Zif268 in the hippocampal DG. The results showed that the proportion of adult-born neurons expressing c-Fos in the exercise groups was significantly higher than that in the sedentary groups, *F*_(__1_,_10__)_ = 21.254, *p* = 0.001. Similarly, the proportion of adult-neurons expressing Zif268 in the exercise groups was also significantly higher than those in the sedentary groups, *F*_(__1_,_10__)_ = 6.985, *p* < 0.05. However, no significant difference was found in the c-Fos or Zif268 expression between the NMT and WMT groups (Figure 9).

**FIGURE 10:**
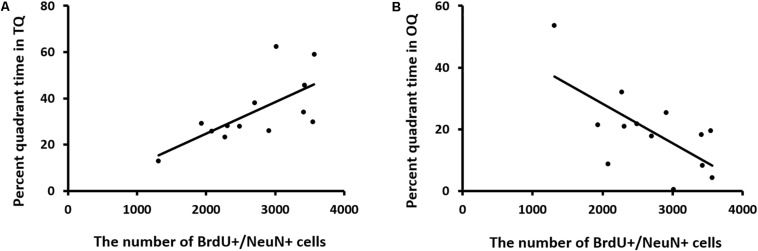
Correlation between neurogenesis and memory retention in reversal learning. **(A,B)** Neurogenesis is positively related to the percentage of time spent in the target quadrant (TQ), while it is negatively related to the time spent in the opposite quadrant (OQ). Data were analyzed using Pearson correlation analysis.

We also analyzed the relationship between neurogenesis and behavioral performance (percent quadrant time in the TQ and OQ during the reversal probe test) using Pearson correlation analysis. A positive correlation was found between neurogenesis and the time mice spent in the TQ (Pearson correlation coefficient, *r* = 0.676; *n* = 13), whereas a negative correlation was found between neurogenesis and the time mice spent in the OQ (Pearson correlation coefficient, *r* = −0.662; *n* = 13) (Figure 10).

## Discussion

Previous studies have shown that exercise facilitates forgetting but enhances reversal learning by decreasing PI (Epp et al., 2016). PI, a type of memory interference, usually occurs when consolidated memories inhibit current learning. However, the effect of old memories on new learning is not only negative, it can also be positive, such as in proactive facilitation, which refers to old memories enhancing new learning when the content of the new and old memories overlaps. In the present study, we did not find PI but found proactive facilitation effects of previous memory traces on spatial learning in both the exercise and sedentary groups. Although there was a decrease in memory retention in the exercise group, the positive impact of the previous experience on the subsequent new learning was preserved. An indirect search strategy might have survived from the previous experience to facilitate new learning. In addition, exercise enhanced the learning performance in both the NMT and WMT groups. This facilitation effect might result from a higher activation of adult-born neurons.

It is widely accepted that exercise facilitates learning and memory. However, most of the studies showing this facilitation have focused on the effects of exercise on the performance of learning tasks following the exercise, whereas few studies have investigated the effects of exercise on the memories consolidated before the exercise. Studies on neurogenesis-induced forgetting have indicated that exercise facilitates the forgetting of previous memory traces through neurogenesis (Akers et al., 2014; Gheorghe et al., 2018). Epp et al. (2016) further suggested that neurogenesis-induced forgetting might reduce PI from remote memory traces when learning items with overlapping information (Epp et al., 2016). Our results were consistent with these previous studies, showing that exercise enhanced the forgetting of remote memories. In our study, we found an increased latency to find the platform and a decreased number of crossings in the target platform location after 4 weeks’ delay in the WMT + Ex group, whereas no significant changes were found in the WMT + Sed group. More crossings in the TQ were also found in the WMT + Sed group when compared with those in the WMT + Ex group. In addition, the number of crossings in the TQ were significantly higher than that in the other quadrants in the WMT + Sed group. These data indicated that, to some extent, there was a decrease in memory retention for mice in the WMT + Ex group, while the previous memory traces were retained for mice in the WMT + Sed group. Conflicting data were also found when examining memory retention in the WMT + Sed group, which showed no difference in the percent quadrant time among the different quadrants during the recall test. The non-preferred exploration in the TQ for mice in the WMT + Sed group might have been because the probe test during recall was the second probe test without a platform in the pool. Thus, although these animals remembered the position of the platform and explored that location immediately after entering the pool, when they did not find the platform where they expected it to be, they might have been unsure whether it was still in that quadrant and therefore explored the other quadrants as well. In addition, we did not find that exercise decreased PI when we investigated the effects of exercise-induced forgetting on new learning in a similar paradigm. Indeed, no PI from previous memory traces was found when mice underwent new learning during the reversal training. In the present study, we conducted post-exercise training of mice with or without remote memory traces. When we compared these groups after the spatial learning task, we found that the experience of the mice that had been exposed to the MWM task facilitated the associative learning in the reversal spatial task. This facilitation was observed for experienced mice in both the exercise and sedentary groups. It should be pointed out that although significantly lower memory retention was found in the WMT + Ex group, the experience of pre-exercise training equally facilitated the acquisition of the new platform location during reversal learning. These results indicated that the retention of the platform location might be unnecessary for the subsequent associated learning. The strategy learned from experience might be the key factor to benefit new learning. Previous studies have shown the facilitation of proactive experience on later associative learning for both motor and episodic memory tasks (Verneau et al., 2015; Zimmermann et al., 2016). This kind of positive transfer makes acquiring information more efficient. We suggest that the strategies obtained from experience survived exercise-induced forgetting. These strategies remained to benefit the subsequent new learning. To understand the search strategies during reversal learning, we examined Whishaw’s index during the reversal probe test. The data showed that mice in the exercise groups spent more time in the corridor connecting the start position with the platform before the first entry in the platform location, and there was no difference between the NMT + Ex and WNT + Ex groups. This indicated that exercise facilitated the development of a direct search strategy during water maze learning. However, the surviving strategies from the previous experience might be indirect search strategies. The type of search strategies used will require further study. Another study using paired-associate learning to investigate the benefit of neurogenesis-induced forgetting on learning of conflicting information found that the neurogenesis-induced forgetting decreased PI in new learning with content that was highly conflicting, but no effect was found for learning of information with low conflict interference (Epp et al., 2016). In our study, we utilized a classic spatial learning paradigm, the MWM, to explore the effects of exercise-induced forgetting on new learning (reversal learning) using the same water maze. We found enhanced facilitation but not reduced interference from the remote spatial learning memory. The inconsistency between the results of the previous study and ours might be because of the different levels of difficulty of these two tasks. A study by Epp et al. (2016) exposed mice to two contexts (e.g., context A: white container; context B: striated container) with one of two scented beddings (e.g., odor 1: coffee; odor 2: cinnamon). Mice responded to specific context-odor pairs to gain rewards (e.g., rewarded for responding to odor 1 in context A and responding to odor 2 in context B). During reversal learning with high interference, the rewarded pairs were reversed (i.e., rewarded for responding to odor 2 in context A and to odor 1 in context B). Based on the hippocampal cognitive map theory, each context corresponds to a specific hippocampal map (environmental cues influence behavior), and “remapping” occurs when the context changes (Kubie et al., 2019). Therefore, there are two maps in the paired-associate task and two “remapping” processes during reversal learning. However, in the present study, there was only one hippocampal cognitive map during the acquisition of the MWM task, and one “remapping” process during reversal learning in the MWM. As a result, during new learning of additional conflicting information in the paired-associate task, memory traces may have provided more interference than facilitation. In addition, as previous studies have shown, swimming-induced stress might have a negative effect on cognitive performance (Shen et al., 2019). Thus, the poorer performance of mice in the NMT group during the acquisition of post-exercise spatial learning might, to some extent, result from swimming-induced stress. Exercise-induced enhancement in swimming speed was found in the NMT groups. Therefore, whether the decreased latency in the NMT + Ex group was affected by the increased swimming speed is unclear. Despite this, we can assert the beneficial effect of exercise and experience on new learning through the results of the path length, which is not affected by swimming speed. In addition, we found that exercise increased exploration in the TQ during the probe test in the NMT groups. These results are consistent with previous studies that have shown the beneficial effects of exercise on memory retention (Park et al., 2018; Leem et al., 2019).

Computer model results have suggested that the integration of adult-born neurons into a neural circuit results in neurogenesis-induced forgetting (Weisz and Argibay, 2012; Aimone, 2016). New adult-born neurons integrating into a synaptic circuit might either replace or coexist with the existing synapse, which can lead to forgetting of consolidated memories (Finnegan and Becker, 2015). In addition, neurons that are newly born at certain stages (about 3–4 weeks in mice) have special characteristics that lead them to be more sensitive than mature neurons during memory encoding. Researchers have reported that immature neurons of mice between 1 and 1.5 months of age express more *N*-methyl D-aspartate receptor subtype 2B (NR2B) receptors, which play a main role in neuroplasticity (Ge et al., 2007). Furthermore, less sensitivity to inhibitory interneurons has been found in adult-born neurons (Malleret et al., 2010). Therefore, neurogenesis is considered to be the main neuroplasticity factor mediating the enhancement of cognitive function after exercise. In the present study, we investigated the participation of adult-born neurons in post-exercise learning by labeling cells with antibodies to BrdU, NeuN, and IEGs. As shown in several previous studies, increased neurogenesis was found in the hippocampus of mice in the exercise groups. In addition, we found a greater proportion of newborn neurons positive for c-Fos and Zif268 in the exercise groups than in the sedentary groups, indicating increased activation of these newborn neurons when obtaining a spatial memory in mice that exercised. A previous study has shown that early-age exercise enhances the activity of adult-born neurons in the hippocampal DG after learning (Shevtsova et al., 2017). Other researchers also find that running exercise facilitates memory encoding by reorganizing the adult-born neurons (Vivar et al., 2016). However, there are conflicting results showing that neurogenesis has no effects on spatial learning in rats (Groves et al., 2013). Controversial findings also appear in studies examining the effect of neurogenesis on memory retention. Numerous studies in mice have shown the facilitation of neurogenesis on forgetting by using spatial memory or fear conditioning tasks (Akers et al., 2014; Epp et al., 2016), whereas Kodali et al. (2016) found no effect of neurogenesis on forgetting. Such results might suggest differential effects of neurogenesis on various types of cognition (e.g., spatial learning) among different species. In our study, we investigated the association between neurogenesis and memory retention during reversal learning. We found a positive correlation between neurogenesis and the time mice spent in the TQ and a negative correlation between neurogenesis and the time mice spent in the OQ during the reversal probe test. However, because correlation does not necessarily imply causation, it is unclear whether neurogenesis contributed to the changes in memory retention. In addition, our results showed that the activity of the newborn neurons was not different between groups with or without previous memory traces, which might suggest that adult-born neurons might equally participate in memory encoding whether in a similar situation or a totally novel context. Considering the results of a study by Bostock et al. (1991), a given representation in the hippocampus reliably reactivates when animals return to the corresponding environment. Pignatelli et al. (2019) suggested that successful recall of a contextual memory is influenced by the activation of the same engram cells activated during memory encoding. Such evidence provides a basis for the assertion that a similar group of engram cells would be activated when animals return to the same environment. Therefore, neurons activated during the probe test would involve the same group of engram cells activated during learning in water maze tasks.

During reversal learning for the WMT groups, we found a lingering exploration of the former TQ in the sedentary group, which might explain the lower learning efficiency in the sedentary group when compared with the exercise group. During reversal learning, hippocampal remapping occurs, and another set of place cells is activated (Kubie et al., 2019). Adult-born neurons are more sensitive than mature neurons in responding to stimulation (Beining et al., 2017). This might lead to the preferred involvement of the adult-born neurons in hippocampal remapping. The participation of newborn neurons in hippocampal remapping benefits pattern separation, a process distinguishing overlapping contextual representations. Thus, mice in the exercise group, which had more adult-born neurons, may have more easily distinguished the changed context during reversal learning and more rapidly achieved new memories.

In summary, our study explored the proactive facilitation effect of diminished spatial memory induced by exercise on the acquisition of a subsequent related memory. We found that although the previous memory traces were weakened after 4 weeks of exercise, the previous experience still increased the efficiency of new learning in a similar paradigm. Further investigation of the search strategies involved in spatial learning indicated that previous experience might have provided indirect search strategies for subsequent related new learning, and these search strategies were not affected by exercise intervention. Exercise facilitated learning efficiency through the acquisition of a direct search strategy. We also investigated the participation of newborn neurons in new learning between animals with or without a similar experience and found that the activation was equal. However, to what extent these newborn neurons participated in this process and whether the existing mature neurons participated in this process will require further study.

## Data Availability Statement

The datasets are available on request to any qualified researcher. The raw data supporting the conclusions of this article will be made available by the authors.

## Ethics Statement

All animal experiments were ethically reviewed, approved, and conducted according to the animal care guidelines of the Ethics Committee of Shanghai University of Sport.

## Author Contributions

CZ and RL were responsible for the study concept and design and provided critical revision of the manuscript for important intellectual content. CL contributed to the research conduction and drafted the manuscript. All authors critically reviewed the content and approved the final version for publication.

## Conflict of Interest

The authors declare that the research was conducted in the absence of any commercial or financial relationships that could be construed as a potential conflict of interest.

## Footnotes

1 http://www.anymaze.co.uk
